# Rethinking Vulnerability as a Radically Ethical Device: Ethical Vulnerability Analysis and the EU’s “Migration Crisis”

**DOI:** 10.1007/s12142-023-00685-5

**Published:** 2023-06-14

**Authors:** Sylvie Da Lomba, Saskia Vermeylen

**Affiliations:** Law School, University of Strathclyde, Lord Hope Building, Level 3, 141 St James Road, Glasgow, G4 0LT Scotland, UK

**Keywords:** Ethical vulnerability analysis, Hospitality, EU asylum and migration policies, Migration crisis, Ukraine, Temporary protection

## Abstract

We reinvigorate vulnerability theory as a radically ethical device — ethical vulnerability analysis. We bring together fuller vulnerability analysis as theorized by Fineman and Grear in conversation with Levinas and Derrida’s radical vulnerability and the ethics of hospitality to construct a theoretical framework that is firmly anchored in the realities of the everyday that are vulnerability and migration. This novel framework offers a thinking space to subvert approaches to migrants and migration as it compels us to come face-to face with the “other”, which in turn renders the political accountable by her.

We deploy ethical vulnerability analysis to deconstruct the EU’s “migration crisis” and investigate whether the activation of temporary protection for displaced persons from Ukraine signifies a humanizing turn in the EU’s asylum and migration policies. In this regard, we submit that this hospitable moment constitutes an “exception to the rule” rather than a paradigm shift.

## Introduction

In this paper, we reinvigorate vulnerability theory as a radically ethical device — ethical vulnerability analysis. We bring together fuller vulnerability analysis as theorized by Fineman and Grear in conversation with Levinas and Derrida’s radical vulnerability and the ethics of hospitality to construct a theoretical framework that is firmly anchored in the realities of the everyday that are vulnerability (Fineman 2008; Turner 2006) and migration (Chetail 2019, p. 16). We posit that ethical vulnerability analysis offers a thinking space to subvert how we consider migrants and migration as it compels us to come face-to face with the so-called “other”, which in turn renders the political accountable by the lived human experience. Accordingly, we demonstrate how ethical vulnerability analysis demands that we confront and reimagine the EU’s response to migrants and migration.

To date, the language of vulnerability in the EU’s migration and asylum policies has done little to recognize and respond to migrants’ vulnerability; rather, we witness an instrumentalization of vulnerability. Similarly, the language of “crisis” participates in the problematizing and securitization of migrants. Both the language of vulnerability and crisis serves to support policy objectives entrenched in the “traditional relationship of migration law and the nation”, which closely links migration law to the sovereign power and national identity and consequently construes migration as an anomaly (Dauvergne 2004). This, in turn, makes for policy objectives that are detached from the fundamentally human realities that are vulnerability and migration.

As we deconstruct the EU’s “migration crisis” through the lens of ethical vulnerability analysis, we investigate whether its response to the plight of people who have fled Ukraine marks a hospitable and humanizing turn in the EU’s asylum and migration policies. In this regard, we submit that the activation of temporary protection^1^ for displaced persons from Ukraine constitutes an “exception to the rule” rather than a paradigm shift. Indeed, we point out that the EU’s welcome is not extended to those the EU regard as being “too different”.

Against this backdrop, we reclaim the concept of vulnerability and argue that ethical vulnerability analysis provides a potent critical device to investigate and respond to the construction of the EU’s “migration” crisis. We posit that this crisis^2^ is, in reality, a hospitality and humanity crisis. We contend that it is born out of the EU and its Member States’ failure to open the door to the other (Levinas 1969; Derrida 2000), which makes for fundamentally inhuman and inhumane policies.

We start this paper by discussing how the language of vulnerability and crisis plays out in the EU’s migration and asylum policies. We then demonstrate how fuller and radical vulnerability analyses enlightened by Levinas and Derrida’s radical vulnerability and ethics of hospitality offer a fundamentally ethical version of fuller vulnerability analysis. Having set out our novel ethical vulnerability analysis, we show how it can humanize the EU’s asylum and migration policies. We finish the paper with concluding remarks on how ethical vulnerability analysis can radically transform the EU’s migration and asylum policies.

## The EU’s “Migration Crisis”: A Hospitality and Humanity Crisis

We posit that the EU has not faced and is not facing a “migration crisis”.^3^ We contend that what the EU has been experiencing is a violent crisis of hospitality and, ultimately, a violent crisis of humanity. We argue that this crisis is of the EU’s own making; it is caused by its asylum and migration policies’ failure to recognize and respond to the fundamentally human experiences that are vulnerability and migration. We further submit that the EU’s remarkably swift response to the international protection needs of people fleeing Ukraine^4^ cannot of itself humanize its asylum and migration policies since inhospitable tenets such as deterrence continue to underpin these policies (Phillips in this issue).

The EU’s “migration crisis” was framed from the European perspective. It is the increase in the numbers of refugees who reached the EU territory in 2015 that spurred the EU and its Member States to recalibrate these migration flows as a “crisis”.^5^ Over 911,000 refugees and migrants arrived on European shores in 2015; “[o]ver 75 per cent (…) had fled conflict and persecution in Syria, Afghanistan, or Iraq.” (Spindler 2015) The construction of the EU’s “migration crisis” can be described as a decontextualized “reflection of numbers” (Crawley 2016, p. 13). The crisis narrative developed by the EU and its Members States ignores the realities of migration in our “uneven globalised world” (Grear 2013, pp. 41 and 53). Indeed, the increase they experienced “pale[d] into insignificance relative to the number of refugees in other countries outside” the EU (Crawley 2016, p. 13). As Gilbert put it, the response of the EU, and especially the response of its Member States, to what was an acute need for international protection was an “overreaction of [the then] twenty-eight states that [were] still among the wealthiest on the planet” (Gilbert 2015, p. 534). Bello observes that “migration” crises are “socially constructed scattered inflamed reactions that have been happening since the end of the Cold War, as a consequence of forced movements of people that a variety of conflicts and instabilities have produced across the planet” (Bello 2020, p. 1327).

Critically, the term crisis is particularly susceptible to instrumentalization (Charlesworth 2002). It does not say who is responsible, nor what can be done. Importantly, the alarm that comes with the language of crisis resonates with the EU’s restrictive policy agenda in the fields of asylum and migration. Thus, rather than elicit a humanizing of the EU’s approach, the framing of heightened protection needs as a “migration crisis” has served to entrench inhospitality deeper in its asylum and migration policies. Similarly, the global health crisis caused by the COVID-19 pandemic “has been seized as an opportunity to strengthen existing deterrence measures and hamper migrants’ access to asylum” (Tazzioli and Stierl 2021, p. 539).

As we underscore in the introduction, the language of vulnerability is also instrumentalized. Language can of course be instrumentalized to serve “good” causes; however, in the case of the EU’s asylum and migration policies and their ubiquitous usage of the language of vulnerability, it has not. Quite the contrary, we contend that vulnerability has been misconceptualized and misappropriated to serve these policies’ inhospitable objectives.

EU laws and policies in matters of asylum and migration affix the label “vulnerable” to certain migrants such as minors, unaccompanied minors, disabled people, [and] elderly people’.^6^ This approach — the vulnerable group approach — is not peculiar to EU laws and policies.^7^ Its proponents postulate that it presents two key advantages. They argue that — unlike universal vulnerability — the vulnerable group approach recognizes that some of us are more vulnerable and thus in need of (greater) protection.^8^ We fully accept this viewpoint as true to the human experience. What we object to is that, whilst it affirms the particular dimension of vulnerability, the vulnerable group approach eschews its universality. This is not without risks for those whose vulnerability is ignored. Revealingly, EU laws and policies do not ascribe the label “vulnerable” to young male adult migrants who are commonly deemed “invulnerable”; yet they are undeniably vulnerable to migration policies (Sözer 2021, p 15). Conversely and importantly, universal vulnerability recognizes both dimensions (Fineman 2008, p. 10). Hence, while this approach accepts that some migrants are inevitably more vulnerable than others, its baseline is that all are vulnerable as human beings. Thus, and in contrast to the vulnerable group approach, the notion of universal vulnerability compels EU laws and policies to regard all migrants as vulnerable, including “unwanted” migrants. Advocates of the vulnerable group approach further posit that it makes the notion of vulnerability more concrete and consequently more useful (Levine et al. 2004, p. 45). Yet, its deployment in asylum and migration policies produces distorted vulnerability narratives that are shaped by policy objectives. Critically, these reconstructed narratives — and consequently, the language of vulnerability — conceal migrants’ vulnerability to the EU’s policies. “Much of the suffering and death that we witness at Europe’s borders is indeed the result of European border policies” (Palister-Wilkins 2016, p. 312). Troublingly, measures which participate in the violence inflicted on migrants, such as pushbacks (Phillips in this issue), are presented as vulnerability-sensitive initiatives, designed to protect migrants’ lives, when their primary aim is to police the EU’s external borders and keep the other out. It is true that migrants’ vulnerability cannot solely be attributed to migration laws and policies; for example, they are inevitably vulnerable as embodied beings (Fineman 2008, p. 9). However, it is equally true that their resilience to these other vulnerabilities is undermined by hostile migration and asylum policies. For example, it is well established that the Common European Asylum System does not adequately uphold the rights of asylum seekers with disabilities (O’Sullivan and Ferri 2020).

Atak et al. further observe how “the term “vulnerability” too often serves to portray migrants in a negative light, as helpless victims” (Atak et al. 2018, p. 2). This observation resonates with Butler’s point that the “vulnerable” is “fixed in a political position of powerlessness and lack of agency” (Butler 2016, p. 25). Importantly, because the vulnerable group approach reduces vulnerability to something entirely negative (Maiani 2020), it obscures its generative dimension (Fineman 2012, p. 96). This, in turn, contributes to the problematizing of migrants’ dependency on others — even though we are all dependent on others (Herring 2011, p 256). This is apparent in the characterization of asylum seekers as a burden, which accounts for the lack of solidarity within the internal and external dimensions of the Dublin system (Moreno-Lax 2017, p. 762).^9^ Yet, as we demonstrate below, the generative dimension of human vulnerability is essential to forging the relationships that we need to live (Fineman 2012, p. 96), including our relationship with the other. We further show how ethical vulnerability analysis humanizes the host’s rapport to the other’s dependency as it de-problematizes it and recognizes the other’s role in responding to the host’s own dependency on others. Put it differently, ethical vulnerability analysis recognizes the host and guest’s interdependence as a reality of the human experience. It follows that our approach fully accepts migrants as partakers in the distribution — but also the creation — of resilience-building resources in the host state. As explained below, migrants’ access to such resources remains contingent on their particular vulnerability, but the latter is no longer reconstructed in light of the immigration status they have been assigned by the host state. Furthermore, the EU’s migration and asylum policies establish a hierarchy based on the Other’s “degree of otherness”. As we stress below in relation to displacement from Ukraine, (perceived) closeness to or alignment with the “European us” opens doors — doors that are hardly ajar to those deemed “too different”. From an ethical vulnerability analysis, this is a highly problematic practice. As we argue in the section on radical vulnerability, for the EU’s migration and asylum policies to be ethical, they must open the EU’s borders to those who are “different”. To be fully hospitable, the EU must open its borders to the “radical Other” such as young Muslim men. As we argue in Sect. 4, while the EU uses the other’s strangeness as a justification to close its borders, radical vulnerability theory subverts this stance by precisely using the other’s alienness as an ethical demand to open borders.

Crucially, the language of vulnerability employed in the EU’s asylum and migration policies is not the protective tool that it purports to be. This language is routinely deployed as a mere rhetorical tool. For example, inadequate protection in EU laws and policies for unaccompanied migrant children (Sedmak et al. 2017) and asylum seekers with disabilities (O’Sullivan, Ferri 2020) remains a concern, notwithstanding their categorization as “vulnerable”. Tellingly, the Commission’s Proposal for an Asylum and Management Regulation unnecessarily prolongs procedures for determining the Member State responsible for examining unaccompanied children’s asylum claims,^10^ which would constitute an unwelcome overturning of the Court of Justice of the EU’s case law.^11^

The EU’s response to the plight of persons displaced from Ukraine begs the question of whether its (in)hospitality must be reassessed. As over 5 million persons have fled Ukraine (UNHCR 2022) — the largest flow of refugees in Europe since the Second World War (Shotter 2022) − , the EU has promptly — and for the first time — activated temporary protection.^12^ Strikingly, the language of “refugee crisis” and “migration crisis” has not, for the time being, marred the EU’s discourse; nor has the language of vulnerability been deployed at the expense of these persons. The Decision on Temporary Protection does not employ the concept of vulnerability, but the Commission’s guidance stresses that “[d]ue consideration should (…) be given to the particular needs of vulnerable persons and children”.^13^ Below, however, we argue that this hospitable moment does not signify a humanizing turn in the EU’s asylum and migration policies. Indeed, the EU’s present welcome is not extended to the “more other” Other, making the Union’s hospitality towards certain persons fleeing Ukraine an “exception to the rule” rather than a paradigm shift. It is important to acknowledge at this juncture that, whilst the EU is to date “more hospitable’ to Ukrainian refugees, there is emerging evidence that the welcome in EU Member States may well be waning (Ambrose 2022; Lawley 2022, p. 4).

## The Construction of Ethical Vulnerability Analysis

Above, we level strong criticism at the language of vulnerability deployed in the EU’s migration and asylum policies; we deplore its misconceptualization and instrumentalization in pursuit of hostile policy objectives. However, this rebuke must not be interpreted as a rejection of the concept of vulnerability — quite the contrary. We reclaim universal vulnerability and construct a novel theorization of vulnerability. The latter offers a multi-tier approach which brings together Grear’s fuller vulnerability analysis — which expands Fineman’s state-centric vulnerability analysis — and Levinas and Derrida’s radical vulnerability theory, to create a potent and ethical critical device to investigate and respond to human vulnerability, the “primal human condition” (Fineman 2015, p. 614). We further show how Levinas and Derrida’s ethics of hospitality buttress the ethical drive that comes with our conceptualization of vulnerability as they transform our relationship with the other.

## Fuller and Radical Vulnerability Analyses

We argue that, in contrast with the vulnerable group approach, fuller vulnerability analysis offers a potent critical device to recognize and respond to human vulnerability in all its complexities and diversity. Two distinctive features explain the powerfulness of fuller vulnerability analysis. Firstly, it conceptualizes vulnerability in light of the human experience. Secondly, because it is grounded in the realities of our uneven globalized world, fuller vulnerability analysis compels us to reconsider how we look at and responds to the vulnerability and dependency of the other. Fuller vulnerability analysis thus prompts a radical rethink of our rapport to human vulnerability and ensuing dependency on others which, in turn, reinvigorates responses to our shared vulnerability.

Fuller vulnerability analysis recognizes that vulnerability is universal and constant (Fineman 2008, p. 1); it acknowledges that we are all vulnerable at all times, both as embodied (Fineman 2008, p. 9) and socially embedded beings (Grear 2013, p. 49). The theorization of vulnerability as universal, however, does not eschew its particular dimension; we all experience vulnerability in different ways because of our “different forms of embodiment” and circumstances (Fineman 2010–11, p. 269). Fuller vulnerability analysis’s all-embracing conceptualization of vulnerability is also apparent in its affirmation of its generative dimension. It is undeniable that vulnerability has negative traits; however, it also possesses a defining positive dimension that is born out of our reliance on resilience-building resources. It is our vulnerability and resultant dependency on others that bring “opportunities for innovation and growth, creativity, and fulfillment. It makes us reach out to others, form relationships, and build institutions” (Fineman 2012, p. 96).

Critically, fuller vulnerability analysis works towards resilience-building; it does not countenance invulnerability as an aim to be pursued (Fineman 2010–11, p. 269). Vulnerability analysis convincingly debunks the myth of the invulnerable liberal subject and replaces this fictional subject with the vulnerable subject — the human subject (Fineman 2008, pp. 10–12). The demise of the fictional invulnerable subject can have a transformative effect on the EU’s migration and asylum policies. As we point out above in relation to the treatment of young male adult migrants, the recognition of universal vulnerability that underpins the affirmation of the vulnerable subject eschews the very idea of “invulnerable” migrants. Below, we further show how this uncompromising rebuke of the invulnerable means embracing our dependency on others as a fundamentally human trait, which in turn can de-problematize migrants’ dependency on the host.

We concur with Fineman that resilience-building calls for a responsive state (Fineman 2010–11, p. 251) as the latter plays a vital role in the creation and distribution of resilience-building resources. However, unlike Fineman, we situate the state within its global setting; indeed, the state is one actor amongst many and the complex power relationships that form the fabric of our globalized world significantly mediate its ability to respond to vulnerability (Grear 2013, pp. 41 and 55). For example, it is imperative that enquiries into the EU’s asylum and migration policies take account of power imbalances and wealth inequalities between EU Member States and (some) third countries. For this reason, we espouse Grear’s fuller iteration of vulnerability analysis as it firmly locates investigations into vulnerability within the “systems of power and privilege that interact to produce the webs of advantages and disadvantages” within our unequal, interdependent and interlocked world (Grear 2013, p. 55).

Furthermore, and contrary to Fineman’s vulnerability theory, fuller vulnerability analysis denationalizes the vulnerable subject, which is critical to extending hospitality to the other. For Fineman, the responsive state’s duty is essentially owed to citizens, though she hints that this duty may extend to “others to whom [the State] owes some obligation” (Fineman 2010–11, p. 256). Fuller vulnerability analysis, importantly, looks beyond the state-citizen relationship as it situates the vulnerable subject within her uneven globalized world. Consequently, fuller vulnerability analysis is well equipped to engage with the realities of international migration as it reclaims its fundamentally human nature and recognizes its transnational dimension. It anchors enquiries into migrants’ vulnerability in the realities of our world, which is crucial to building the resilience of both “guests” and “hosts”. Critically, fuller vulnerability analysis subverts the bounded state-centered baseline at the heart of migration laws and policies that sees the nomadic being as an anomaly who must be controlled — a baseline that perpetuates policy failings as the EU’s experience shows (Lavanex 2018, p. 1195). Because it denationalizes the vulnerable subject and firmly locates her within our uneven globalized world, fuller vulnerability analysis enables us to relate to the other’s vulnerability and dependency as a fellow vulnerable human being. Importantly, this also means that fuller vulnerability analysis enables us to reclaim vulnerability as a foundation of human rights law (Da Lomba 2019). The rationale for this conceptualization of vulnerability lies in its universality (Turner 2006, p. 28) as well as in its relevance to all human rights (Grear 2010, p. 135). In their reflection on vulnerability’s relationship with human rights, Timmer and others argue that vulnerability ‘offers an opportunity to re-interrogate the role of human rights as key constraints on state power and catalysts for social change’ (Timmer et al. 2021, p.191). They notably observe how the concept of vulnerability has been instrumental in furthering the protection of migrants’ social rights, albeit with limitations (Timmer et al. 2021). Critically, and in contrast with our vulnerability framework, theirs does not envisage the denationalization of the vulnerable subject. Yet, the denationalization that comes with fuller vulnerability analysis is precisely what severs the link between one’s recognition as a fully-fledged human rights subject and one’s legal status (Da Lomba 2019). As it reconstructs the human right subject in line with the universal premise of human rights, fuller vulnerability analysis can extend protections to those migrants currently confined to the margins of human rights protection regimes (Dembour and Kelly 2011). As we show below, this is key to humanizing the EU’s migration and asylum policies. It follows that fuller vulnerability analysis prompts a “deeply ethical impulse” that empowers us “to envision cooperations and solidarities across the divide and the asymmetry” of our globalized world (Radhakrishnan 2003, p. vii) as it extends the reach of our emotional identification with others (Carens 1996, p. 156).

The construction of ethical vulnerability analysis requires that we cement the inclusiveness that comes with fuller vulnerability analysis. We do so through the deployment of radical vulnerability analysis and the ethics of hospitality developed by Levinas in conversation with Derrida. We posit that these consolidate fuller vulnerability analysis’ ethical catalyst as it deepens our responsibility towards the other.

## Radical Vulnerability Analysis

In this part of the paper, we develop the idea of radical vulnerability to subvert the way in which the EU’s migration and asylum policies have used the “crisis” to close borders and prevent the mobility of migrants. Through radical vulnerability, we inscribe new meaning to existing concepts such as hospitality and responsibility by engaging with Levinas’s ethics of responsibility and Derrida’s ethical and political response to the “crisis” of the stranger at the border. In agreement with Costello’s observation that the EU’s handling of the “migration crisis” has led to more stringent restrictions (Costello 2020), we seek to show how this crisis — the EU’s hospitality and humanity crisis — can also be the catalyst for profound and meaningful change. Crisis narratives can indeed have a positive influence in fast-tracking a genuine change in laws and policies. However, as Costello rightly reminds us, crises can also engender policy paralysis (Costello 2020). In the same vein, Authers and Charlesworth observe that crises can jumpstart but also overwhelm regulatory capacities to the extent that it paralyses any further action (Authers and Charlesworth 2013, p. 20). For instance, the EU’s “migration crisis” has undeniably served to bolster what we see as illegal obstacles to migration. Yet, just like other citizens, refugees, and other migrants should be offered the opportunity to benefit from transnational mobility. Critically, we develop the notion of radical vulnerability to buttress the argument in support of human mobility. Indeed, radical vulnerability makes our vulnerability analysis profoundly ethical, which, in turn, injects a transformative ethical element into migration and asylum policies, including the EU’s.

Our ethical vulnerability analysis is not unique in demanding that we open the door in response to “the deaths at sea, dangerous journeys, and the comprehensive illegalization of refugee travel” (Costello 2020, p. 22). However, our approach is innovative in that it compels us to investigate how crisis as atrocity can provoke an ethical obligation to redirect the course of migration and asylum policies from containment to hospitality. In doing so, our ethical vulnerability analysis puts the spotlight on the vulnerability of the face of the other as a commanding trope for change in migration and asylum policies. Significantly, the change in question makes hospitality the defining quality of these policies.

The photograph of Alan Kurdi, a 3-year-old Syrian Kurdish boy who was found drowned on a Turkish beach on 2 September 2015 as he and his family tried to reach the Greek Island of Kos became a visual testimony that urgent political action was needed, even though many migrants had already died when attempting the dangerous crossing in the Mediterranean (Adler-Nissen et al. 2020, pp. 75–76). Like other European state leaders, the then UK Prime Minister David Cameron expressed sorrow; he also declared that “Britain [was] a moral nation and we fulfil[ed] our moral responsibilities” (Dathan 2015). However, as we all know too well, taking up that moral responsibility never materialized in a concerted effort to open up the European borders. It is true that the death of Alan as well as events in Hungary^14^ had prompted Germany to suspend the Dublin system and open its borders to refugees and migrants (Futák-Campbell and Pütz 2022, p. 62). This policy, however, was reversed in July 2018 (France 24 with AFP 2018). Besides, by March 2016, the EU-Turkey joint statement designed to limit the number of refugees reaching Europe had been struck;^15^ a deal, Angela Merkel the then German Chancellor, had played a significant role in bringing about (Futák-Campbell and Pütz 2022, p. 62).

From the perspective of Levinas’s radical ethical theory, however, Alan’s lifeless body becomes precisely the “site” where vulnerability is releasing an ethical responsibility (Levinas 1969). Levinas draws on his very personal experience of the Holocaust^16^ to situate vulnerability in the face-to-face encounter between the self and the other. While fuller vulnerability analysis mobilizes the asymmetry of our globalized world to evoke empathy towards the other, Levinas goes further and compellingly argues that it is the other’s destituteness and vulnerability that approach us from a height that provoke the feeling of responsibility (Levinas 1969, p. 66). In other words, to call upon responsibility, Levinas situates the ethical encounter in the face of the other and uses the naked face as a trope to instigate vulnerability which, in turn obliges responsibility (Levinas 1989b, p. 48). The nakedness of the face signifies that the self, when encountering the destituteness of the other,^17^ has no other option but to respond to the needs of the person who is facing her (Levinas 1969, pp. 245–246). This is an an-archaic command that emanates from the destitution of the face of the other (Levinas 1969, pp. 198–199 and 245–246). Thus, in Levinas’s ethics, vulnerability transcends the proposition of emotional identification with others, and instead, acts as a catalyst for the introduction of an infinite responsibility towards the vulnerable. Critically, this means that this command exists prior to the promulgation of any moral rule, law or sovereignty of the state. This, in turn, makes Levinas’s ethics particularly valuable within the context of migration at it compels us to respond to refugees and other migrants’ vulnerability.

Turning again to the photograph of Alan Kurdi’s frail and limp body on the beach, we posit that, by zooming into his vulnerability through Levinas’s ethics, we can reinvigorate the impulse to seek radical change in crisis. From a Levinasian perspective, Alan’s lifeless body represents a profound command of facilitating mobility, opening borders, and offering hospitality to all those whose vulnerable face carries a duty for us to respond to. Thus, radical vulnerability analysis as enlightened by Levinas’s ethics of responsibility requires that responses to crises embrace the human, which subverts and humanizes the EU’s response to migration and vulnerability.

The EU’s response to displacement from Ukraine compels us to ask whether the Union is finally shouldering its responsibility towards the other. Below, we argue that we are witnessing a hospitable moment rather than a humanizing shift. Indeed, while the EU is (presently) proving responsive to the international protection needs of some — “more different” others — are not extended the same hospitality. The UN High Commissioner for Refugees has commented on “the ugly reality that some Black and Brown people fleeing Ukraine (…] have not received the same treatment” and lamented the “persistent reports of unequal or discriminatory treatment” of non-Ukrainians refugees in Europe (Grandi 2022). The story of twice refugee Jehad Kaware illustrates that not all fleeing Ukraine are welcome in Europe (Pop 2022). Jehad fled the Gaza Strip in 2017 and ended up seeking asylum in Ukraine; because of the war there, he had to flee again. The “welcome” that this non-European refugee received in Brussels is strikingly different from that extended to Ukrainian refugees despite their fleeing the same war and atrocities. While Ukrainian refugees must simply register at a center for Ukrainian nationals, Jehad had to report to an asylum office and thus faced the same inhospitable conditions that many other refugees have had to experience since 2015. Moreover, Jehad could only find lodging in a makeshift shelter which he shared with Jordanians and Nigerians when Ukrainian nationals received, upon their arrival in Belgium, a SIM card, food and housing that was swiftly arranged due to Belgian families opening their door to Ukrainian refugees.

Having bolstered the ethical dimension of fuller vulnerability analysis through the deployment of radical vulnerability analysis, we engage with Levinas’s ethics of hospitality in conversation with Derrida’s. We demonstrate how these heighten our responsibility towards the other, which strengthens fuller vulnerability analysis’ ethical core.

## Ethics of Hospitality

At this juncture, we engage with Levinas’s and Derrida’s ethics of hospitality to consolidate the host’s responsibility towards the other and strengthen the ethical dimension of our vulnerability theory. The significance of the concept of hospitality in this context has been acknowledged by other authors (Aliu and Aliu 2022). We posit that this responsibility must take the form of an unconditional welcome that demand that the other’s vulnerability and consequently her humanness be acknowledged through naming the other as a person of flesh and blood. Significantly, giving the other a “face” acts as a reminder that migration laws must respond to and are to be held accountable by the migrant. As we have argued elsewhere in more detail, radical vulnerability analysis has also implications for how the state must regulate migration and how indeed radical vulnerability analysis can hold legislators and the law accountable to act ethically towards migrants. To summarize our argument briefly, according to a Levinasian ethics, the inter-human relation that is guided by an unconditional responsibility towards the other (in this case, migrants) also shapes the condition for ethical behavior on the part of the state and its migration laws and policies. Levinas situates vulnerability in the triangular relationship between the vulnerability of the subject, the other and the state (Mao 2020, p. 211). Although Levinas’s theory of vulnerability starts with the encounter of the other who demands of us (as individuals) to act responsibly towards their requests for hospitality, because of the triangular relationship between the self, the other and the state, this demand also extends to the state that, in its migration policies, must also respond to this original demand for hospitality by the other. There is an explicit call from Levinas that any state institution — including the law — must give an unconditional welcome to the “Other” (Levinas 1998a, b, p. 159).

From a Levinasian and Derridean perspective, the problem with current migration laws lies with their having erased any trace of the vulnerability of the other, which makes for laws that are oblivious to the migrant experience and support restrictions to hospitality. Indeed, the moment unconditional hospitality is turned into migration laws, a hostile space is being created — a space with its own rules and norms that no longer offers unbounded hospitality to the guest (Dufourmantelle 2013, p. 15). This is what precisely happened in the summer and autumn of 2015. In the face of heightened vulnerability, the EU bolted its doors even more securely to protect itself against the “influx” of newcomers portrayed as a threat. A good example of this securing is the renaming and transformation of FRONTEX into a fully-fledged European Border and Coast Guard as part of the EU’s response to the “migration crisis” (Guiraudon 2018, p. 151).^18^

According to Derrida, Levinas’s welcoming of the face in totality and infinity can be interpreted as an act of hospitality (Derrida 1999, p. 21). In his eulogy for Levinas, Derrida points to Levinas’s use of *la porte* in totality and infinity as a trope for hospitality (Derrida 1999, p. 26). For Derrida, the open door signifies reaching out to the symbols of hospitality: “with one’s hand held out, addressing oneself to the other so as to give him something to eat or drink” (Derrida 1999, p. 26). However, for Derrida, the open door also represents the welcoming of the infinite responsibility towards the other, which stands for justice (Derrida 1999, p. 23; Levinas 1969, p. 93). The relationship with the other is not an abstract relationship that exists outside the world of experience; it demands that we share the world or indeed offer hospitality (Derrida 1969, 156). Opening the home to hospitality is a sign of what is meaningful in life. Critically, Levinas reminds us that our obligation to offer hospitality to the other predates our encounter with her.

However, as Derrida so aptly shows, hospitality is a rather ambiguous concept. The ambivalence is already contained in the etymological roots of the Latin word *hostis* which includes the words “inviting master” and “invited guest”, but also refers to hostility and the enemy. Reflecting on the shared Latin root for host and hostility, Derrida uses the word *hosti-pitalité* to represent this ambiguity (Derrida 2001, p. 7). In order to develop hospitable migration and asylum policies there are two requirements that need to be fulfilled.

First, Derrida underscores that welcoming the guest also means to differentiate between the foreigner and the absolute other. This distinction relates to the wider paradox Derrida exposes between conditional and absolute or unconditional hospitality. He stresses that unconditional hospitality “breaks with the law of hospitality as right or duty, with the pact of hospitality” (Derrida 2000, pp. 25–27). Indeed, for unconditional hospitality to happen, the door must be opened not only for the other who possesses the traits that the guest requires, but also to the anonymous other (Derrida 2000, p. 25). To put it differently, hospitality must be extended to the absolute foreigner, the one with no status (Derrida 2000, p. 25). To foreshadow already what this would imply within the context of the EU’s migration and asylum policies from Derrida’s perspective, these policies could only live up to the scrutiny of unconditional hospitality — and by extension be perceived as ethical — when a generous and unconditional welcome is extended to the absolute Other. According to Derrida, this requires that priority be given to the ultimate foreigner who, in this case, can be exemplified by young Muslim men. However, in order for unconditional hospitality to materialize, it is also necessary to subvert the host–guest relationship.

Critically, Derrida’s argument therefore reinforces Levinas’s idea that hospitality is allowing the Other to come into our own being or, as Dufourmantelle phrases it, “the transcendent quality of hospitality (…) [is] urging to give shelter to the other within our self” (Dufourmantelle 2013, p. 22). It follows that from a Derridean perspective, there can only be “true” or unconditional hospitality when the radical vulnerability of the other is welcomed to such an extent that the distinction between hosts and guests disappears (Cheah 2013, p. 70). Significantly, in Derrida’s unconditional hospitality, the guest is not the only vulnerable being; the host is also vulnerable. Her vulnerability stems from her responsibility to open her house to any stranger without, however, having the power to lay down the rules and conditions of her hospitality (Cheah 2013, pp. 71–72). Importantly, this preparedness to be vulnerable on the part of the host requires an alienation from the home, the nation-state and sovereignty (Cheah 2013, p. 73). As we elaborate below both from a theoretical standpoint and then more concretely, we construe the exigencies of unconditional hospitality as a defining and enduring aim of migration policies. As we illustrate below, this aim must serve as the humanizing compass that guides the constant reshaping of the EU’s asylum and migration policies.

However, while Derrida is calling for unconditional hospitality, it is also clear that, from a practical viewpoint, this is unfeasible to achieve in political and legal terms. Derrida suggests that there can be no choice between conditional and unconditional hospitality as the two concepts exist together in paradoxical relations (Derrida 2003, p. 129). It may thus seem that Derrida is asking the impossible, as he argues that migration laws must use the aporia between conditional and unconditional hospitality. However, we follow Zaccaria’s proposition that this aporia consists of finding a new political and juridical climate that transforms the welcome of the other from visitation to invitation (Zaccaria 2013, pp. 182–184). For example, prompted by European and national appeals, we are witnessing many Europeans opening their homes to Ukrainians fleeing the war. However, as Wabel postulates, appealing to the moral obligation for European citizens to “step it up” and offer shelter to those in need is not enough (Wabel 2020, p. 57). Accordingly, we contend that hospitality and generosity must be embedded in migration laws and policies.

In sum, our theoretical framework — ethical vulnerability analysis — is grounded in the lived experience. Critically, this is what empowers our theory to change migration laws and policies into hospitality. Ethical vulnerability analysis brings together fuller and radical vulnerability analyses and the ethics of hospitality to yield an ethical turn that transforms relationships between hosts and guests. Importantly, our ethical obligation towards others calls for generous and asymmetric responses to their vulnerability that go beyond enabling their mere survival — living is not surviving. Consequently, in addition to subverting relationships between hosts and others, the proposed framework reassesses the relationships between ethics and the political as it renders the political accountable to the demands of the ethics of hospitality.

## Humanizing the EU’s Asylum and Migration Policies

In this section, we demonstrate how ethical vulnerability analysis subverts the inhospitable tenets of the EU’s migration and asylum policies. It does so by anchoring these policies in the lived human experience. Before we set out how ethical vulnerability analysis humanizes the EU’s relationship with the other, we critique the EU’s proclaimed humanizing turn.

The European Commission’s 2020 New Pact on Migration and Asylum talks about a “fresh start”^19^ and opens with a declaration by the Commission President, Ursula von der Leyen, in which she declares that “[w]e will take a human and humane approach” to migration and asylum.^20^ This suggests a humanizing turn of the EU’s asylum and migration policies, but the Commission’s package of proposals on migration and asylum dashes hopes of a meaningful rethink of these policies. These proposals essentially attempt to relaunch phase three of the Common European Asylum System (CEAS) (Peers 2020). It is not uncommon for crises to stifle change. Costello observes that, “[a]lthough crises may sometimes serve as a catalyst for deep change (…), equally they can distract from structural problems or even generate policy paralysis. Regrettably, such paralysis is precisely what is currently evident in the EU” (Costello 2020, p. 21). The construction of the EU’s “migration crisis” has inhibited reform of its asylum and migration policies as it consolidates *realpolitik* (Thym 2020) and its restrictive baseline — its “inevitable” problematizing of migrants and migration — as the only conceivable response*.*

In the absence of a profound rethink of the EU’s asylum and migration policies, deterrence strategies (Phillips in this issue; Aleinikoff 2018, p. 612) that see in asylum seekers and other unwanted migrants a burden that must be “shifted” to third countries, continue to be developed. Growing reliance on measures such as pushbacks and interdiction combined with the (re)surfacing of ideas of containment and externalization are creating a regime of *non-entrée*. This regime is in part responsible for thousands of deaths in the Mediterranean and other egregious violations of international law, including breaches of the fundamental principle of non-refoulement (Lang and Nagy 2021). For example, grave human rights breaches have occurred as a result of agreements concluded between the EU and third countries, notably Turkey and Libya (Nakache and Losier 2017; Phillips in this issue). These cooperation frameworks were adopted in the wake of the EU’s “migration crisis”.^21^ Both purported to provide a quick response to this “crisis”, but their primary aim was to reduce irregular migration in the EU (Thevenin 2021, p. 465). Moreno Lax aptly refers to the EU-Turkey deal as an example of “external non-solidarity” (Moreno-Lax 2017, pp. 759–761), an agreement which is symptomatic of an acute solidarity crisis that corrupts the EU’s asylum and migration policies.

The EU’s response to the international protection needs of displaced persons from Ukraine, however, begs the question of whether we are finally witnessing the promised “fresh start”. On 4 March 2022, the Council of the EU agreed to activate temporary protection for Ukrainian citizens and (certain) others fleeing the war in Ukraine.^22^ Denmark, which is not bound by EU asylum and immigration law due to its opt-out, “introduced a special law that strongly resembles [the EU’s temporary protection framework] directive”.^23^ This development is remarkable on two counts: first, its swiftness — the decision was adopted just over a week after Russia launched its invasion of Ukraine; and secondly, the fact that temporary protection was activated at all. Indeed, the 2001 Temporary Protection Directive^24^ had never been implemented and its repeal was on the EU’s agenda.^25^ Yet, in a matter of days, temporary protection became the cornerstone of the EU’s answer to cross-border displacement from Ukraine. There is not space to analyze the Decision on Temporary Protection in detail, but three key features can be highlighted. Protection is immediate and there is no application process,^26^ which enables beneficiaries to bypass protracted asylum procedures. Protection is initially for one year with the possibility of extending it for up to 3 years.^27^ Temporary protection comes with a range of rights, entitlement to a residence permit and access to the labor market, housing, social welfare and medical care, and to education for children.^28^

There is no doubt that the prompt activation of temporary protection constitutes a most welcome, vital, humane response to the human tragedy unfolding in Ukraine. However, it also raises very troubling questions as to the EU’s double standards. The EU’s response to the plight of those fleeing Ukraine and Syria makes for very contrasting tales. In February 2015, the Commission decided against putting forward a proposal for the activation of temporary protection for those fleeing the conflict in Syria and the ensuing “crisis” in the Mediterranean.^29^ The Commission, inter alia, stressed that EU Member States were concerned that granting temporary protection would create another “pull factor”.^30^ Mentioning Italy as an example, the Commission further noted that the Member States’ asylum systems were still working, one of the objectives of temporary protection being to 'alleviate pressure on national asylum systems’ (EU Council 2022). Yet, a few months later, the Council of the EU agreed a temporary and exceptional relocation mechanism from Italy and Greece to other Member States of persons in need of international protection as these two Member States’ asylum systems were no longer “coping”.^31^ Paradoxically but tellingly, whilst numbers — in 2015, more than 911,000 refugees and migrants had arrived on European shores (Spindler 2015) — were deemed too low to warrant the activation of temporary protection, they were considered high enough to level the “pull factor” argument at its possible deployment. Numbers were also considered high enough to warrant the label of “crisis” and render the EU and its Member States vulnerable to Syrian and other “others”. How can we explain why similar arguments were not levied at the activation of temporary protection for persons who have fled Ukraine? The enormity and proximity of the war in Ukraine partly explain the EU’s readiness to open its door. More cynically — but relatedly — one could surmise that Ukraine’s geographical location precludes the EU from shifting its responsibilities to third countries. More disturbingly, however, we submit that the EU’s hospitality has much to do with the *Europeanness* of the “typical refugee” from Ukraine. To put it differently, we postulate that, because “they look like us”, the EU and its Member States are not so much opening their door to “strangers” than letting in “quasi-us”. For example, the Visegrád Four’s (Hungary, Poland, the Czech Republic, and Slovakia) hospitality towards Ukrainians who have fled the war stand in great contrast to their rejection of the EU’s emergency relocation scheme for refugees^32^ and unwillingness to host Muslim refugees (Bocskor 2018; Krzyżanowska and Krzyżanowski 2018). Wabel, drawing on Simmel’s work (Simmel 2013), explains how the other’s strangeness and alienness is being used to protect EU’s membership. According to the logic of Simmel, the other as stranger has the intention to stay. This opens up the possibility that the stranger has the potential to become an EU citizen. This provokes fear and resentment, but their intensity varies with the stranger’s degree of (dis)similarity. The more distant the stranger becomes from the members of the community, the greater the fear and resentment raised by the prospect of her inclusion in the community (Wabel 2020, p 61). This inclusion–exclusion logic is apparent in the EU’s contrasted responses to the plight of Syrian and Ukrainian refugees. While the former are perceived as the Muslim other and therefore more different from the European “us”, the latter are perceived to be more similar because they share a commonality in religion, culture, and history.

Importantly, the point we make in respect of the EU and its Member States’ selective hospitality finds support in the Decision on Temporary Protection. Those who have fled Ukraine are treated differently depending on their nationality and immigration status, and not all are beneficiaries. Temporary protection applies both to Ukrainian nationals and to stateless persons and nationals from other non-EU countries who were granted international protection or “equivalent national protection” in Ukraine (Article 2(1)(a) and (b) of the Decision on Temporary Protection). Protection is extended to both groups’ family members (*ibid*, Article 2(1)(c))^33^ and both must “have been displaced from Ukraine on or after 24 February 2022” (*ibid*, Article 2(1)(a) and (b)). Protection is also granted to stateless persons and nationals from other non-EU countries who “were legally residing in Ukraine before 24 February 2022 (…) and who are unable to return in safe and durable conditions to their country or region of origin” (*ibid*, Article 2(2)). Protection, however, is not extended to their family members, although the Commission encourages Member States to do so.^34^ Moreover, protection is not conferred on those third country nationals who resided legally in Ukraine on a short-time basis, such as students or seasonal workers, whether or not they are able to return to their country of origin.^35^ Although EU Member States may extend protection to additional categories of displaced persons from Ukraine (Article 7(1) of Directive 2001/55/EC), it remains the case that a number of foreign nationals who lived in Ukraine and had to flee may find themselves protectionless and in limbo. This is, for example, the case for irregular migrants who resided in Ukraine.^36^ Furthermore, there is “emerging evidence of inconsistent practices and racial profiling at border crossings (e.g., in Hungary and Poland) with non-Ukrainians [and stateless people] facing barriers to accessing the territory [of the EU]” (Nash 2022). It is also the case that foreign nationals “without proof of permanent residence or international protection in Ukraine may need to apply for asylum or another form of protection according to the laws of their host country” (Nash 2022). Nash points out that “this could pose serious issues in terms of their access to rights and services and their ability to obtain protection, if denied access to asylum procedures (e.g., as is the case in Hungary)” (Nash 2022).

The recent activation of temporary protection was vitally timely, but the EU’s welcome to the other is selective. As we have just observed, EU’s hospitality does not extend equally — if at all — to all displaced from Ukraine. Furthermore, the “more other” other continues to experience the EU’s inhospitality. For example, migrant pushbacks at the EU’s external borders persist and Greece continues to reject asylum claims lodged by people who have transited through Turkey on the basis that it is a safe third country — though “Turkey has not accepted any readmissions since March 2020” (Meijers Committee 2022). Presently, there is no indication that the hospitality extended to (some of) those fleeing Ukraine is part of a “hospitality awakening”, that would signify the “fresh start” heralded by the 2020 New Pact^37^ or indeed changing EU migration and asylum policies into something that would resemble unconditional hospitality. This would require opening the EU’s borders to all people fleeing from the conflict in Ukraine. Rather, the EU’s hospitable moment begs the unsettling question of whether the EU’s welcoming of those who “look like us” from Ukraine will mean greater hostility towards the “more other” Other. From a human rights perspective, unconditional hospitality requires that all humans — irrespective of their migration status — be given what they need to live as cohabitees. Ultimately, it is from this secure and safe position that the one who was guest can become host and in turn offer generosity to the other. Despite unconditional hospitality being a rather abstract and theoretical proposition, its practical value for policy is rather simple. Migration laws and policies can be deemed “successful” when they enable the one who was once the guest to make a home that can host new guests in the future.

Despite all the shortcomings of the EU’s asylum and migration policies that we have identified in this paper, we are still of the opinion that, paradoxically, the EU’s ethos can provide a solid basis for the development of “human and humane” asylum and migration policies as long as it is framed in light of the notion of unconditional hospitality. According to Waldenfels, this requires embracing the “alien” aspect of the other and allowing the normality to be shaken (Waldenfels 2011). We must thus accept that the guest has capacity to challenge the status quo or indeed “redefine one’s own sense of belonging” (Wabel 2020, p. 68). Significantly, the fears that the other may disrupt normality can be turned into something positive as it draws attention to the fact that the host lives in a state of abundance. It is from this secured and emboldened feeling of sovereignty and security that an opening can be created to give unconditional hospitality to the other, including the very distant other such as the Syrian refugee. This also caters for allowing and welcoming the guest’s ambiguous feelings towards the other. By being aware that the other will never belong and will always be at the periphery of normality, we are compelled to keep on questioning our own sense of belonging, our identity and society’s identity. “The alien call which draws us into the alien experience comes from elsewhere; there is something ex-territorial in it, which also means that it de-territorializes ourselves” (Waldenfels 2011, p. 80). This means that the other has the capacity to question our own sense of belonging, with the consequence that the other’s experience is extended to those who thought they belonged. Hence, the other questions our own sense of belonging so that we all share an “in-between sphere”, a non-belonging in the belonging (Waldenfels 2011, p. 80). Instead of fighting the other through stringent migration and asylum policies, or through making a distinction between those who look (more) like us (Ukrainians) and those we perceive as (more) different (for example, Syrians), the Other “yields the insight that nobody is […] entirely at home in this world” (Waldenfels 2011, p. 81).

To link this to the problematic of the EU’s migration and asylum policies, Article 2 of the Treaty on European Union provides that the Union is founded, inter alia, on the values of respect for human dignity and respect for human rights and emphasizes that such values are critical to societies that uphold solidarity. We contend that the EU’s ethos and ethical vulnerability analysis can speak to one another. To be more precise, ethical vulnerability analysis has the potential to reinvigorate the EU’s ethos as it renders the EU accountable by the other. with the other firmly located at the heart of the European project, her humanity becomes the commanding core who defines the EU’s asylum and migration policies.

With their outlook on migrants and migration radically transformed and, more broadly, their understanding of what it means to be human, the EU and its Member States are compelled to discharge fully their international obligations, notably their human rights and international refugee law obligations. The deployment of ethical vulnerability analysis thus gives substance to the EU’s unfulfilled commitment to ensuring “full respect for the principle of non-refoulement and fundamental rights.”^38^ Critically, our approach also subverts human rights’ relationship with the other. Above, we observe how ethical vulnerability analysis enables us to reclaim vulnerability as a foundation of human rights law and consequently affirm the vulnerable subject — the human subject — as the bearer of human rights. In particular, as it denationalizes the vulnerable subject, ethical vulnerability analysis recognizes migrants as fully-fledged human rights subjects. Therefore, our theorization of vulnerability humanizes human rights (further) as it firmly locates migrants within human rights protection regimes and, in doing so, bolsters the humanizing of the EU’s asylum and migration policies. In other words, as it strives to decouple human right protection from one’s status in the nation-state, ethical vulnerability analysis provides these policies with an underpinning that supports hospitality. Importantly, humane migration and asylum policies further demand that the EU and its Member States reassess these policies’ external dimension. Indeed, ethical vulnerability analysis requires that these policies account for our uneven globalized world and thus cease to “shift” the duty that the EU and its Member States owe to the other to countries outside the EU, commonly countries which find themselves in a power imbalance. In other words, in addition to recognizing and responding to the other’s vulnerability, humane EU migration and asylum policies must account for the (greater) vulnerability of other members of the international community. Again, this idea is not alien to EU law. Article 21(1) of the Treaty on European Union provides that “[t]he Union's action on the international scene shall be guided by the principles which have inspired its own creation, development and enlargement, and which it seeks to advance in the wider world” — including “the universality and indivisibility of human rights and fundamental freedoms, [and] respect for human dignity”. Article 21(1) further states that “[t]he Union shall seek to develop relations and build partnerships with third countries (…) which share [its] principles”. Importantly, as it recalibrates the EU and its Member States’ relationships with the other and non-EU actors, ethical vulnerability analysis does away with much deplored aspects of their current migration and asylum policies such as pushbacks and externalization.

Critically, ethical vulnerability sees the humanizing of the EU’s migration and asylum policies as instrumental in the wider and deeper humanizing of the EU’s, its Member States’, their institutions’ and, importantly, their communities’ relationship with the other. As we stress above, our approach demands that the host embraces the other as a cohabitee and host in the making. As we further underscore, this means making ourselves vulnerable to her and accepting some form of alienation as we make our home the other’s home too. For example, in addition to requiring that reception conditions for asylum seekers in the EU fully uphold human rights obligations, ethical vulnerability analysis commands that the EU host accepts asylum seekers as fellow co-habitees rather than “parasitic guests” — fellow cohabitees whose vulnerability and ensuing dependency on others are embraced as inherent human traits. Elsewhere, we demonstrate how ethical vulnerability analysis empowers “guests to affirm their claim to social welfare as the hosts’ co-habitees”, thereby obligating the former to share what may be limited resources.

Ethical vulnerability analysis means that hospitality towards the other can become the rule in the EU as opposed to a “hospitable gesture” reserved to those “who look like us”. We recognize that our approach calls for an uncompromising reimagining of the EU’s migration and asylum policies. As mentioned above, we also accept that we may never achieve unconditional hospitality. This, however, does not mean that ethical vulnerability analysis cannot shape the political. Quite the contrary, our approach provides an ethical compass that makes humanizing the EU’s migration and asylum policies their defining objective and hospitality their primary aim. We posit that ethical vulnerability analysis offers a humanizing way out of cycles of aborted and failing reforms of the EU’s asylum and migration policies and a way forward that gives meaning to the European Commission’s commitment that the EU “will take a human and humane approach” to migration and asylum.^39^

## Some Concluding Remarks

In this paper, we have subverted the meaning of vulnerability and how it has been used in the construction of the EU’s “migration crisis”. We have developed the concept of (radical) ethical vulnerability analysis by putting Fineman’s vulnerability analysis and Grear’s fuller vulnerability analysis into conversation with Levinas’s and Derrida’s radical vulnerability and ethics of hospitality. This has allowed us to dislodge the entrenched practice of instrumentalizing vulnerability as a deterrent to opening borders and positioning the EU, its Member States and their citizens as being vulnerable vis-à-vis new arrivals. With migrants constructed and perceived as the other, their vulnerability remains hidden behind veils of unwillingness to engage with their lived experience. This has created a politics of impasse best described as a deep reluctance to find a humane solution to what has been presented erroneously by the EU as a “migration crisis”. Levinas’s ethics of radical vulnerability has given us the language and tools to see the crisis in the face of the other — a hospitality and a humanity crisis. His ethics which is based on seeing, first, the hardship in the face of the other, and second, letting this hardship command us to respond to her vulnerability, has allowed us to embed an ethical obligation at the heart of migration and asylum laws and policies. This ethical obligation manifests itself in the gesture of welcoming and offering hospitality to the new arrivals. By linking radical vulnerability to an ethics of hospitality, we are able to reinstall the importance of mobility at the heart of migration. We conclude that the “migration crisis” narrative has led to an entrenched situation of closing borders, which deprives migrants from the vital resilience-building resource that is mobility — a resource that can make the difference between life and death.

It follows that ethical vulnerability analysis empowers us to reimagine the EU’s asylum and migration policies in light of the realities of the human experience. As it humanizes the EU’s relationship with the other, it enables the paradigm shift that would make hospitality the rule rather than an exception. The very idea of such a paradigm shift is likely to be dismissed as overly idealistic and thus unrealistic by realists. However, as Finlayson points out, the status quo bias that commonly comes with realism makes it “a conservative force, aimed at clipping the wings of more “idealistic”” thinking (Finlayson 2017, p. 264). It is certainly the case that calls for pragmatism continue to frustrate meaningful reform of the EU’s asylum and migration policies (Karageorgiou 2020). Furthermore, we have pinpointed the schizophrenic approach (Gibney 2004, p.2) of the EU member states to asylum seekers and refugees. Driven by a fear and mistrust of the other, states do not fulfill their responsibilities under international refugee and human rights law. Their obligations are sidelined under the guise of the need to secure their borders and sovereignty (Bigo 2002, p. 65). Drawing upon the work of Hannah Arendt and her call for the existence of the right to have rights (Arendt 1973), Hirsch and Bell argue that to be a human being demands that humanity be extended to those who are in need of international protection. Indeed, to be human is not a law in itself; it is an ontological claim that “entails a right to belonging and participation” to be “guaranteed” by the state (Hirsch and Bell 2017, p. 426). It follows that, by closing their borders, states are also depriving both their citizens and refugees from the status of full human beings, which ultimately poses a threat to the very existence of humanity (Arendt 1973, p. 298). Critically, unless and until the EU has the courage to engage in a radically ethical rethink of its asylum and migration policies, there is a real risk that, as the EU extends a hospitable hand to displaced persons from Ukraine, it will push away the “more different” Other more forcefully in what could develop into a deeply inhumane zero-sum game.

## Footnotes

1 Council Implementing Decision (EU) 2022/382 of 4 March 2022 establishing the existence of a mass influx of displaced persons from Ukraine within the meaning of Article 5 of Directive 2001/55/EC, and having the effect of introducing temporary protection, OJEU, L 71/1, 4.3.2022.

2 The term “migration crisis” is commonly used by the EU institutions (e.g., European Council (2021) EU Migration Policy, 6 January 2021. https://www.consilium.europa.eu/en/policies/migratory-pressures/. Accessed 22 August 2022. This EU’s “migration crisis” is also referred to as the EU or Europe’s “refugee crisis” (e.g. Spindler 2015).

3 Others have contested the framing of the EU’s crisis as a “migration” crisis (see, e.g., Crawley 2016; Gilbert 2015).

4 Council Implementing Decision (EU) 2022/382 of 4 March 2022 establishing the existence of a mass influx of displaced persons from Ukraine within the meaning of Article 5 of Directive 2001/55/EC, and having the effect of introducing temporary protection, OJEU, L 71/1, 4.3.2022.

5 In 2015, over 911,000 refugees and migrants reached the EU territory compared with 77,000 in 2014 (Theodorou 2016), p. 178).

6 Directive 2013/33/EU of the European Parliament and of the Council of 26 June 2013 laying down standards for the reception of applicants for international protection (recast), OJEU L 180/96, Chapter IV Provisions for Vulnerable Persons, Articles 21 and 22. The Directive on common asylum procedural standards makes provision for vulnerable persons and refers to the definition enshrined in the reception conditions Directive (Directive 2013/32/EU of the European Parliament and of the Council of 26 June 2013 on common procedures for granting and withdrawing international protection (recast), OJEU L 180/60, Article 31(7)(b); Directive 2008/115/EC of the European Parliament and of the Council of 16 December 2008 on common standards and procedures in Member States for returning illegally staying third-country nationals, OJEU L 348/98, Article 3(9)); and Directive 2013/32/EU of the European Parliament and of the Council of 26 June 2013 on common procedures for granting and withdrawing international protection (recast), Article 31(7)(b).

7 The vulnerable group approach is employed in a range of fields such as international migration law and has gained momentum in others such as migration.

8 For example, the term “pluralization” is used to describe the adoption of population-specific instruments in international human rights law (Mégret 2008), p. 495).

9 The Dublin system sets out which country is responsible for determining asylum application lodged in the EU.

10 Proposal for a Regulation of the European Parliament and of the Council on asylum and migration management and amending Council Directive (EC) 2003/109 and the proposed Regulation (EU) XXX/XXX [Asylum and Migration Fund], COM(2020) 610 final, Article 15(5).

11 Case C-648/11 *MA, BT and DA v Secretary of State of the Home Department*, EU:C:2013:367, para. 55.

12 Council Implementing Decision (EU) 2022/382 of 4 March 2022 establishing the existence of a mass influx of displaced persons from Ukraine within the meaning of Article 5 of Directive 2001/55/EC, and having the effect of introducing temporary protection, OJEU, L 71/1, 4.3.2022.

13 Communication from the Commission on Operational guidelines for the implementation of Council implementing Decision 2022/382 establishing the existence of a mass influx of displaced persons from Ukraine within the meaning of Article 5 of Directive 2001/55/EC, and having the effect of introducing temporary protection, 21 March 2022, 2022/C 126 I/01, p. 4.

14 There had been numerous reports on ill-treatment faced by refugees and migrants traveling through Hungary, including clashes with the Hungarian border police (Futák-Campbell and Pütz 2022), p. 77).

15 EU-Turkey statement (2016) European Council, Press release, 18 March 2016. https://www.consilium.europa.eu/en/press/press-releases/2016/03/18/eu-turkey-statement/. Accessed 12 August 2022.

16 Although the Holocaust and the horrors of the Nazi regime have left the biggest imprint on Levinas’s philosophy, Levinas also reflects in, for example, Entre Nous, on other “evil” events that have characterized the twentieth century. In addition to the two world wars, he also refers to Stalinism, Hiroshima, the Gulags, and the killing fields in Cambodia (Levinas 1998a), p. 86).

17 Levinas makes a distinction between *autre* and *autrui* which are concepts that cannot easily be translated into English. The translator of *Totality and Infinity* discussed with Levinas the translation of *autrui* and *autre*. With Levinas’s permission this was resolved by referring to *autrui* as other (the personal other, the you) and *autre* as other. For Levinas, there are thus two types of otherness. The other relates to the absolute other with whom I have an ethical relation. We have capitalized other only when we specifically refer to the way Levinas uses other to signify the moment that the self, the ego, has opened itself to the other and is in an ethical relationship with the other. For further details, see Levinas 1969, p. 24.

18 The European Border and Coast Guard Agency, formerly Frontex, was set up in 2004 to manage the EU’s external borders.

19 Commission, Communication from the Commission to the European Parliament, the Council, the European Economic and Social Committee and the Committee of the Regions on a New Pact on Migration and Asylum COM(2020) 609 final, Sect. 1.

20 Commission, Communication from the Commission to the European Parliament, the Council, the European Economic and Social Committee and the Committee of the Regions on a New Pact on Migration and Asylum COM(2020) 609 final, opening quote.

21 The EU-Turkey deal was concluded in March 2016 and the agreement with Libya in February 2017.

22 Council Implementing Decision (EU) 2022/382 of 4 March 2022 establishing the existence of a mass influx of displaced persons from Ukraine within the meaning of Article 5 of Directive 2001/55/EC, and having the effect of introducing temporary protection.

23 European Commission (2022) New Danish law for Ukrainians mirrors EU Temporary Protection Directive, 16 March 2022. https://ec.europa.eu/migrant-integration/news/new-danish-law-ukrainians-mirrors-eu-temporary-protection-directive_en. Accessed 22 August 2022.

24 Council Directive 2001/55/EC of 20 July 2001 on minimum standards for giving temporary protection in the event of a mass influx of displaced persons and on measures promoting a balance of efforts between Member States in receiving such persons and bearing the consequences thereof**,** Official Journal L 212, 7 August 2001, pp. 12–23.

25 European Commission, Study on the Temporary Protection Directive, Final Report, Directorate-General for Migration and Home Affairs, January 2016, p 82. See also: European Commission, Proposal for a Regulation of the European Parliament and of the Council addressing situations of crisis and force majeure in the field of migration and asylum, Brussels, 23.9.2020 COM(2020) 613 final, 2020/0277 (COD), Explanatory Memorandum, Sect. 3.1., p. 10.

26 Communication from the Commission on Operational guidelines for the implementation of Council implementing Decision 2022/382 establishing the existence of a mass influx of displaced persons from Ukraine within the meaning of Article 5 of Directive 2001/55/EC, and having the effect of introducing temporary protection, 21 March 2022, 2022/C 126 I/01, p. 3.

27 Council Implementing Decision (EU) 2022/382 of 4 March 2022 establishing the existence of a mass influx of displaced persons from Ukraine within the meaning of Article 5 of Directive 2001/55/EC, and having the effect of introducing temporary protection, Preamble, recital 4.

28 Council Directive 2001/55/EC of 20 July 2001 on minimum standards for giving temporary protection in the event of a mass influx of displaced persons and on measures promoting a balance of efforts between Member States in receiving such persons and bearing the consequences thereof**,** Official Journal L 212, 7 August 2002, pp. 12–23, Articles 8(1), 12, 13(1), 13(2) and 14(1).

29 European Commission, Study on the Temporary Protection Directive, Final Report, Directorate-General for Migration and Home Affairs, January 2016, p. 19.

30 European Commission, Study on the Temporary Protection Directive, Final Report, Directorate-General for Migration and Home Affairs, January 2016, p. 35.

31 Decision (EU) 2015/1523 [establishing provisional measures in the area of international protection for the benefit of Italy and of Greece, OJ 2015 L 239, p. 146.

32 Court of Justice of the European Union (Grand Chamber), *Slovak Republic and Hungary v Council of the European Union*, Joined Cases C-643/15 and C-647/15, judgment of 6 September 2017.

33 Family members are the protection beneficiary’s spouse or partner (subject to certain conditions), the beneficiary or spouse’s minor unmarried children and other relatives who are dependent on the beneficiary Communication from the Commission on Operational guidelines for the implementation of Council. implementing Decision 2022/382 establishing the existence of a mass influx of displaced persons from Ukraine within the meaning of Article 5 of Directive 2001/55/EC, and having the effect of introducing temporary protection, 21 March 2022, 2022/C 126 I/01, pp. 4–5.

34 Communication from the Commission on Operational guidelines for the implementation of Council implementing Decision 2022/382 establishing the existence of a mass influx of displaced persons from Ukraine within the meaning of Article 5 of Directive 2001/55/EC, and having the effect of introducing temporary protection, 21 March 2022, 2022/C 126 I/01, p. 5.

35 Communication from the Commission on Operational guidelines for the implementation of Council implementing Decision 2022/382 establishing the existence of a mass influx of displaced persons from Ukraine within the meaning of Article 5 of Directive 2001/55/EC, and having the effect of introducing temporary protection, 21 March 2022, 2022/C 126 I/01, p. 6.

36 There were between 37,700 to 60,900 persons in an irregular situation in Ukraine at the end of 2019 (Pozniak et al. 2020), p. 5).

37 Commission, Communication from the Commission to the European Parliament, the Council, the European Economic and Social Committee and the Committee of the Regions on a New Pact on Migration and Asylum COM(2020) 609 final, Sect. 1.

38 Commission, Communication from the Commission to the European Parliament, the Council, the European Economic and Social Committee and the Committee of the Regions on a New Pact on Migration and Asylum COM(2020) 609 final, Sect. 2.1.

39 Commission, Communication from the Commission to the European Parliament, the Council, the European Economic and Social Committee and the Committee of the Regions on a New Pact on Migration and Asylum COM(2020) 609 final, opening quote.
